# Role of Kruppel-Like Factor 5 in Deoxycholic Acid-Mediated Intestinal Transdifferentiation of Esophageal Squamous Epithelium

**DOI:** 10.7150/jca.30050

**Published:** 2019-09-07

**Authors:** Yiju Xia, Yu Fang, Haoxiang Zhang, Caifei Shen, Pu Wang, Wu Yan, Jingwen Li, Yin Xu, Shunzi Shao, Yafei Zhang, Xiaona Yu, Zhihong Peng, Guiyong Peng, Wensheng Chen, Dianchun Fang

**Affiliations:** 1 Department of Gastroenterology, Southwest Hospital, Army Medical University, Chongqing, 400038, P.R. China; 2 Department of Thoracic and Cardiovascular Surgery, The First Affiliated Hospital of Chongqing Medical University, Chongqing 400042, China

**Keywords:** Barrett's esophagus, transdifferentiation, KLF5, deoxycholic acid

## Abstract

Barrett's esophagus (BE) is an acquired condition in which normal squamous epithelium is replaced with metaplastic columnar epithelium as a consequence of gastroesophageal reflux disease. BE is known as a precursor of esophageal adenocarcinoma. Currently, the molecular mechanism underlying epithelial metaplasia in BE patients remains unknown. Therefore, we investigated the role of Krüppel-like factor 5 (KLF5) signaling in the initiation of BE-associated metaplasia. Sprague-Dawley (SD) rats were used to create a surgical model of bile reflux injury. Immunohistochemistry was performed to analyze human and mouse esophageal specimens. Human esophageal squamous epithelial (HET-1A) cells were treated with bile acid and used in transfection experiments. Quantitative real-time PCR and western blot analysis were performed to detect the expression of KLF5, CDX2, MUC2 and villin.

Epithelial tissue from both the rat BE model and human BE patients strongly expressed KLF5, CDX2, MUC2, and villin. Bile acid treatment also increased the expression of KLF5, CDX2, MUC2 and villin in esophageal epithelial cells in a time-dependent manner. Moreover, siRNA-mediated knockdown of KLF5 blocked the expression of CDX2, MUC2 and villin, but transfection of a KLF5 expression vector into esophageal epithelial cells promoted their transdifferentiation into columnar-like cells, as demonstrated by increased expression of the intestinal markers CDX2, MUC2 and villin. Thus, in addition to its function as a transcription factor, KLF5 may be linked to an increased risk of BE development.

## Introduction

Barrett's esophagus (BE), also defined as columnar-lined esophagus, is a metaplastic condition in which the normal non-keratinizing squamous epithelium of the esophagus is replaced by a columnar mucosal epithelium 1,2. The region of columnar esophageal metaplasia is considered the “cancerization field” in which esophageal adenocarcinoma (EAC) develops 3-5. Gastroesophageal reflux disease (GERD) probably plays a major role in the development of BE by inducing repeated mucosal damage. It is widely accepted that chronic GERD leads to inflammation and ulceration of the esophageal squamous mucosa and that persistent and recurrent inflammation and ulceration may lead to columnar metaplasia and, ultimately, intestinal metaplasia. The major harmful components of refluxed gastric material are acid and bile salts. Both clinical and experimental studies have shown that bile acids are noxious to the esophageal mucosa. The severity of mucosal damage is increased in patients who reflux both gastric and duodenal juice compared with patients who reflux gastric juice alone 6. Bile salts, or, more accurately, duodenal content, have been implicated in the pathophysiology of esophageal mucosal injury for decades. Numerous studies have shown significant effects of bile salts and other components of gastroesophageal reflux on cellular physiology, including the activation of protein kinase C and nuclear transcription factors 7. Together with the strong link between GERD and esophageal adenocarcinoma, these findings suggest that bile salts play a role in the pathophysiology of BE and EAC.

Several important developmental signaling pathways and transcription factors have been reported to be critical in inducing transdifferentiation of mature esophageal squamous cells into columnar cells or for causing immature esophageal progenitor cells to undergo columnar, rather than squamous, differentiation (transcommitment) 8. In a rat model of reflux esophagitis, metaplasia develops when bone marrow stem cells enter the bloodstream and settle in the reflux-damaged esophagus 9. Alternatively, studies in mouse models have suggested that metaplasia might result from upward migration of stem cells from the proximal stomach (the gastric cardia) or from proximal expansion of embryonic-type cells at the gastroesophageal junction 10,11. However, it is unclear whether any of these processes contribute to the pathogenesis of BE in humans. Further research is required to fully understand these pathways and their association with known environmental and host risk factors.

Krüppel-like factor 5 (KLF5), also known as intestine-enriched Krüppel-like factor (IKLF), is a zinc finger-containing transcription factor that belongs to the Sp/KLF family of proteins 12. *In vivo* models have demonstrated roles for KLF5 in biological processes such as embryonic development, cardiovascular remodeling, adipogenesis, inflammatory stress responses, and intestinal development 13. KLF5 is expressed in many tissues, where it both activates and represses the transcription of target genes 13. In embryonic stem cells, KLF5 plays an important role in self-renewal and maintenance of pluripotency 14. KLF5 is highly expressed in the gastrointestinal epithelium throughout its development 13. In the adult intestine, KLF5 is localized to highly proliferative cells within intestinal crypts. McConnell et al. 15 demonstrated that the deletion of KLF5 in the postnatal intestinal epithelium disrupted the intestinal crypt architecture and the balance between goblet and enteroendocrine cells within the colon. Bell et al. 16 demonstrated that KLF5 also plays a pivotal role in establishing each villus in the fetal intestine prior to the formation of intestinal crypts. In the absence of villus formation, terminal maturation of small intestinal and colonic cell types was inhibited due to the loss of KLF5. The role of KLF5 in the pathogenesis of BE remains unclear. Our objective for this study was to investigate the role of Krüppel-like factor 5 (KLF5) signaling in the initiation of BE-associated metaplasia. We have demonstrated that deoxycholic acid (DCA)-mediated induction of KLF5 plays an important role in the transdifferentiation of esophageal squamous epithelial cells into an intestinal phenotype and that this event underlies the intestinal metaplasia associated with BE.

## Materials and Methods

### Patients and human samples

A total of 80 patients with GERD-related symptoms, who were diagnosed with endoscopically proven reflux esophagitis (RE, n=59) or BE (n=21, short-segment BE), were enrolled in this study. Of the 21 patients with BE, 17 were men and 4 were women with an age range of 30-60 years (mean age of 42 years at diagnosis). Of the 59 patients with RE, 41 were men and 18 were women with an age range of 30-60 years (mean age of 39 years at diagnosis). After appropriate endoscopy with biopsies, patients were placed in one of three diagnostic classes: 1) BE, 2) esophagitis,3) endoscoped controls. The inclusion criteria for BE was as follows: patients who met the standardized definition of BE, that is, the presence of columnar-lined mucosa in the distal esophagus of any length at endoscopy and the presence of intestinal metaplasia on histologic evaluation 17. All BE specimens lacked either dysplasia or cancerization. Esophageal biopsies were obtained from healthy volunteers (n = 20) serving as normal controls. Fresh endoscopic biopsy specimens were fixed in 10% formalin. Paraffinized sections (4 μm thick) were routinely stained with hematoxylin and eosin. Histological slides were scored blindly and independently by two experienced gastrointestinal pathologists. None of the patients had taken any antibiotics, bismuth compounds, H_2_ blockers or proton-pump inhibitors (PPIs) in the two weeks prior to study entry. The experiments using human materials were approved by the Bioethics Committee of the Southwest Hospital, and signed informed consent was obtained from all patients who participated in the study.

### Animal model and surgical procedures

Seventy eight-week-old male Sprague-Dawley (SD) rats with an average weight of 210 g (±15 g SD) were used in this study. The experiments using animals were approved by the Committee of Experimental Animal Ethics of The Third Military Medical University. All surgeries were performed as described in our previous study 18. (1) In the mixed reflux group (30 rats), the proximal jejunum was laterally anastomosed together with the distal esophagus without gastrectomy. As a consequence, duodenogastroesophageal reflux was induced. (2) In the duodenal reflux group (30 rats), the esophagus was transected at the esophagogastric junction and was anastomosed to the jejunum in an end-to-side manner; then, the stomach was resected, and the proximal duodenum was sutured with plasma muscularis embedding. By this method, duodenoesophageal reflux (without gastric acid) was established. (3) In the control group (10 rats), anesthesia was administered, and mock surgery was performed in which only a skin incision was made and then sutured. The rats in each group were sacrificed at 6 months after surgery. The entire rat esophagus was opened longitudinally and fixed in 10% buffered formalin. Paraffinized sections (4 μm thick) were routinely stained with hematoxylin and eosin for histopathological and immunohistochemical analysis.

### Immunohistochemistry

Immunostaining was performed on paraffinized sections using a microwave-based antigen retrieval technique. The antibodies used in this study included those against KLF5 (1:200), Cdx2 (1:100), MUC2 (1:400) and villin (1:200) (all from Abcam, Cambridge, MA, USA). Sections were treated with an Envision+ DAB kit (Dako, Glostrup, Denmark) according to the manufacturer's instructions. To evaluate the staining intensity, an intensity score (IS) was calculated: no staining intensity was scored as 0, marginal intensity as +1, and strong intensity as +2. The criteria for scoring IHC staining were as follows: Intensity was graded as 0 (negative), 1 (weak), 2 (moderate) or 3 (strong). The proportion of positive cells was graded as 0 (<5%), 1 (5‑25%), 2 (26‑50%), 3 (51‑75%) or 4 (>75%). A final score was derived by multiplying the two primary scores. Final scores of 0‑4 were defined as negative expression (‑), scores of 5‑8 as weak positive expression (+) and scores of 9‑12 as strong positive expression (++) 19. Histological slides were estimated blindly and independently by two experienced gastrointestinal pathologists.

### Cell culture and bile salt exposure

Het-1A cells (a human normal esophageal cell line immortalized via viral SV40 transfection, purchased from ATCC -American Type Culture Collection, USA- in December 2013) at approximately 5×10^5^ cells/ml. Cells were seeded in a 60-mm plastic dish coated with rat-tail tendon collagen type I (Hangzhou Shengyou Biotechnology, China) in complete K-SFM supplemented with 100 U/ml penicillin and 100 U/ml phytomycin to prevent infection. The cultures were maintained at 37°C in a humidified atmosphere at 5% CO_2_ in air. DCA bile salt (50, 100 or 200 μM) (Sigma, St. Louis, MO, USA) diluted in serum-free medium was used. Het-1A cells were cultured in neutral medium (pH 7.2) with or without DCA for 12 hours.

### RNA interference targeting KLF5

Three target shRNA sequences were selected from different regions of human KLF5 (GenBank accession number: NM_00173.4). First, screening was performed to determine the most effective siRNA from 3 siRNAs against KLF5; then, an optimal small hairpin RNA (shRNA) cassette targeting human KLF5 (5'- GCAUCCACUACUGCGAUUACCTT-3') and a non-targeted control shRNA cassette (target sequence 5'-GCACGACTTCTTCAAGTCCTT-3') were cloned into the pGenesil-1 vector (Genesil Biotechnology Co., Ltd., Wuhan, China), which had been digested with BamHI and HindIII. To carry out this experiment, the concentration of 50 nM siRNA-KLF5 and the time point of 48 hours after transfection were chosen for subsequent siRNA experiments as follows. Het-1A cells were incubated in DCA (at 200 µM) for 12 hours after transfection with KLF5 siRNA. The expression levels of KLF5, Cdx2, MUC2 and villin were evaluated via western blot and real-time PCR analyses.

### Lentiviral-mediated over-expression of KLF5

The cDNA sequence encoding full-length human KLF5 (GenBank accession number: NM_00173.4) was amplified via PCR and cloned into the lentiviral vector pGag/Pol (Gene Pharma Co., China) to produce pLV-KLF5. To produce a negative control virus (LV-NC), a short noncoding sequence was cloned into the pGag/Pol vector and processed in parallel with the target gene sequence. Het-1A (3×10^5^) cells were transduced with the lentivirus carrying KLF5 or the noncoding sequence using polybrene (5 µg/ml). At 48 hours after infection, 2 µg/ml puromycin was added to the media to select for lentivirus-infected cells. Real-time PCR and western blot assays were used to detect the expression of KLF5, Cdx2, MUC2 and villin in the stable cell lines.

### Confocal immunofluorescence microscopy

Het-1A cells were fixed with 3.7% paraformaldehyde in PBS for 30 min and were permeabilized with 0.1% Triton X-100 for 10 min at room temperature. After blocking with 1% bovine serum albumin in PBS, the cells were incubated in rabbit anti-human monoclonal antibody serum as previously described. Images were captured using a laser scanning confocal microscope (Olympus- IX70). Optical image sections (0.5 mm) were obtained using Olympus FV500 FluoView Application Software. To compare the expression of KLF5 protein between the control and treated cells, images were acquired using constant acquisition parameters (laser power, confocal aperture, photomultipliers voltage, and gain).

### Quantitative real-time PCR

Cells were collected after stimulation. Total RNA was extracted using Trizol reagent (Gibco BRL), and cDNA was synthesized from 2 µg of total RNA using Superscript reverse transcriptase (Life Technologies, Inc., Carlsbad, CA, USA) as per the manufacturer's instructions. Using the 7500 Real-Time PCR System (Applied Biosystems, Singapore), a real-time fluorescence PCR assay was performed using SYBR Green (Takara Biotechnology, China) and the primers described in Table 1. The relative expression levels were calculated via normalization to the GAPDH gene expression level.

### Protein extraction and western blot analysis

Protein samples were prepared via homogenization of cells in lysis buffer (10 mmol/L Tris-HCl, pH 8.0; 140 mmol/L NaCl; 5 mmol/L EDTA; 0.25 g/L NaN_3_; 10 g/L Triton X-100; 10 g/L deoxycholate; 1 g/L SDS; 0.5 mmol/L PMSF; 1 g/L leupeptin; and 1 g/L aprotinin). The protein concentration was determined using the Coomassie brilliant blue method. Proteins were separated via SDS-PAGE, transferred to PVDF membranes (Millipore, Temecula, CA, USA), blocked with milk/BSA, and then probed with specific antibodies against KLF5 (1:1000), CDX2 (1:800), MUC2 (1:1000), villin (1:1000) (all from Abcam, Cambridge, MA, USA) and GAPDH (1:2000, Kangchen, Shanghai, China). After washing, the blots were incubated in a horseradish peroxidase-conjugated goat anti-mouse secondary antibody, then visualized using an enhanced chemiluminescence reagent (Pierce Biotechnology, Rockford, IL, USA), and subsequently exposed to autoradiographic film. All experiments were reproduced at least three times. The western blotting bands were analyzed using ImageJ software (Bio-Arts, Co., Ltd., Fukuoka, Japan).

### Data analysis

For the data of real-time PCR and western blot, comparisons between groups were assessed via one-way ANOVA followed by Bonferroni's test. The data are expressed as the means ±SEM. For the data from histologic analysis, comparisons were assessed using the t test. Statistical analyses were performed using GraphPad Prism software (GraphPad, San Diego, CA, USA). P < 0.05 was used as the threshold for statistical significance.

## Results

### Gastroesophageal reflux caused increase of KLF5 in human esophageal epithelium

**Figure 1:**
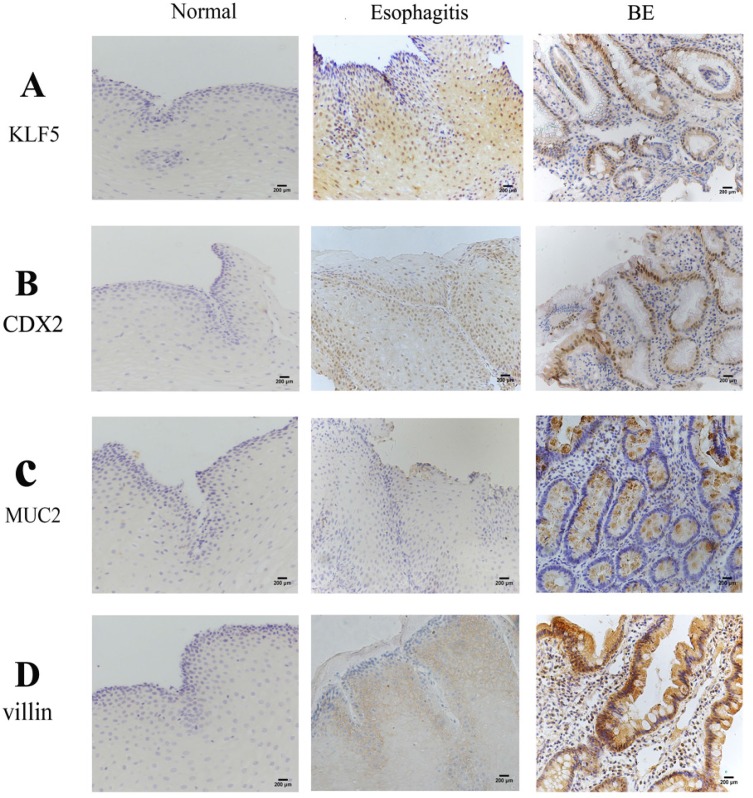
** Immunohistochemistry findings in human esophageal samples.** Representative immunohistochemisty of (A) KLF5; (B) CDX2; (C) MUC2;(D) Villin in human normal.

Examination of KLF5 in patients with BE or esophagitis, and in healthy donors serving as controls, showed that the expression of KLF5 demonstrated a nuclear staining pattern at the site of BE glands. Increased KLF5 expression was observed in BE tissues compared with that in normal esophageal and esophagitis tissues (Fig. 1). As shown in Table 2, substantial nuclear KLF5 staining was observed in 85.7% of BE samples (18/21). In contrast, KLF5 expression was not detected in normal esophageal squamous epithelium (0/20), and weak KLF5 expression (+1) was observed in the basal layers of the squamous epithelium in the esophagitis samples (16/59, 27.1%).

In the assessment of cell lineage distribution, evaluation of the expression of the intestinal markers Cdx2, MUC2 and villin in human BE squamous epithelium revealed increasing expression of Cdx2, MUC2 and villin in the order of normal esophageal, esophagitis and BE tissues. Some esophagitis samples showed weak staining (+1), but no expression was observed in the normal squamous epithelium (Fig. 1C and D). The incidences of positive staining for these markers in BE were significantly higher than those in normal esophagus and esophagitis mucosa (P<0.01).

### Alterations in KLF5 expression in a rat surgical model of bile acid reflux

Among 60 animals in the surgically-generated rat models of duodenoesophageal reflux and gastroduodenoesophageal reflux, 16 (26.7%) died prior to the end of the experiment, including ten from the duodenoesophageal reflux group and six from the gastroduodenoesophageal reflux group. No deaths occurred in the sham-surgery group. Compared with age-matched sham rats, the experimental rats showed a markedly thickened esophagus 6 months after surgery. The major histopathologic changes in both surgical groups are shown in Table 2. BE did not occur in control rats; however, 4 of the 20 (20%) rats in the duodenoesophageal reflux group and 5 of the 24 (20.9%) rats in the duodenogastroesophageal reflux group developed columnar metaplasia with goblet cells, similar to human BE. No significant differences in the incidence of esophageal disease were detected between the duodenoesophageal reflux group and the duodenogastroesophageal reflux group.

**Figure 2:**
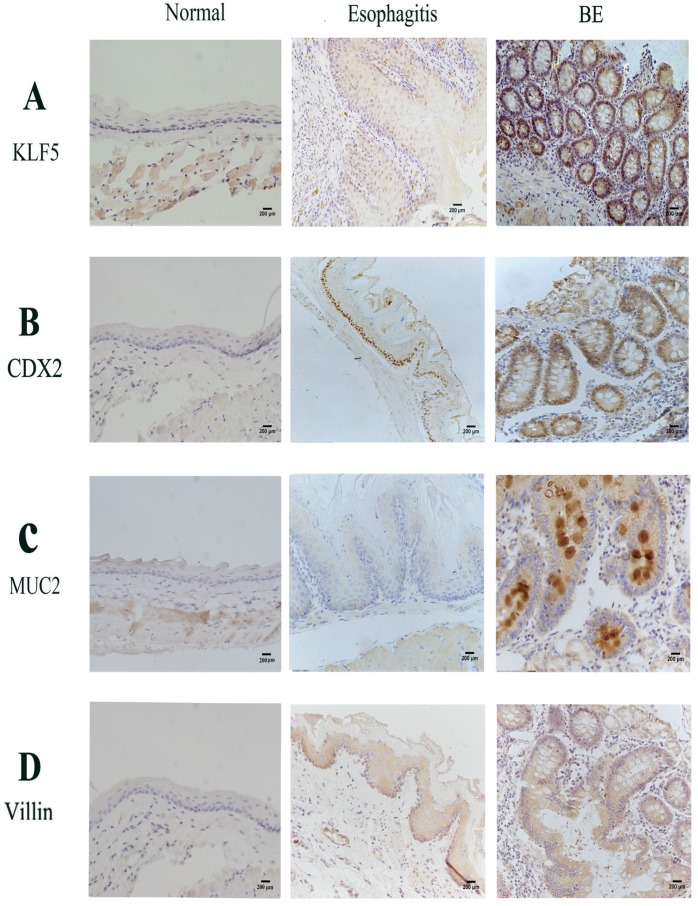
** Immunohistochemistry findings in rats' esophageal samples.** Epithelium was examined 6 months after performance of oesophagealejejunal anastomosis. Representative immunohistochemistry of (A)Klf5; (B) Cdx2; (C) Muc2; (D) Villin in rat normal, reflux esophagitis and BE tissues.

Results of immunohistochemistry studies performed to analyze the expression of KLF5, Cdx2, MUC2 and villin in the rat models showed a marked increase in nuclear staining in BE mucosa compared to that in normal esophageal and esophagitis mucosa (Fig. 2). Similar to human tissue, the rat esophageal tissues showed stepwise increases in the expression of KLF5, Cdx2, MUC2 and villin in the order of normal esophageal, esophagitis and BE squamous epithelium based on immunohistochemical analysis.

### Bile acid exposure induces differential gene expression in Het-1A cells

**Figure 3:**
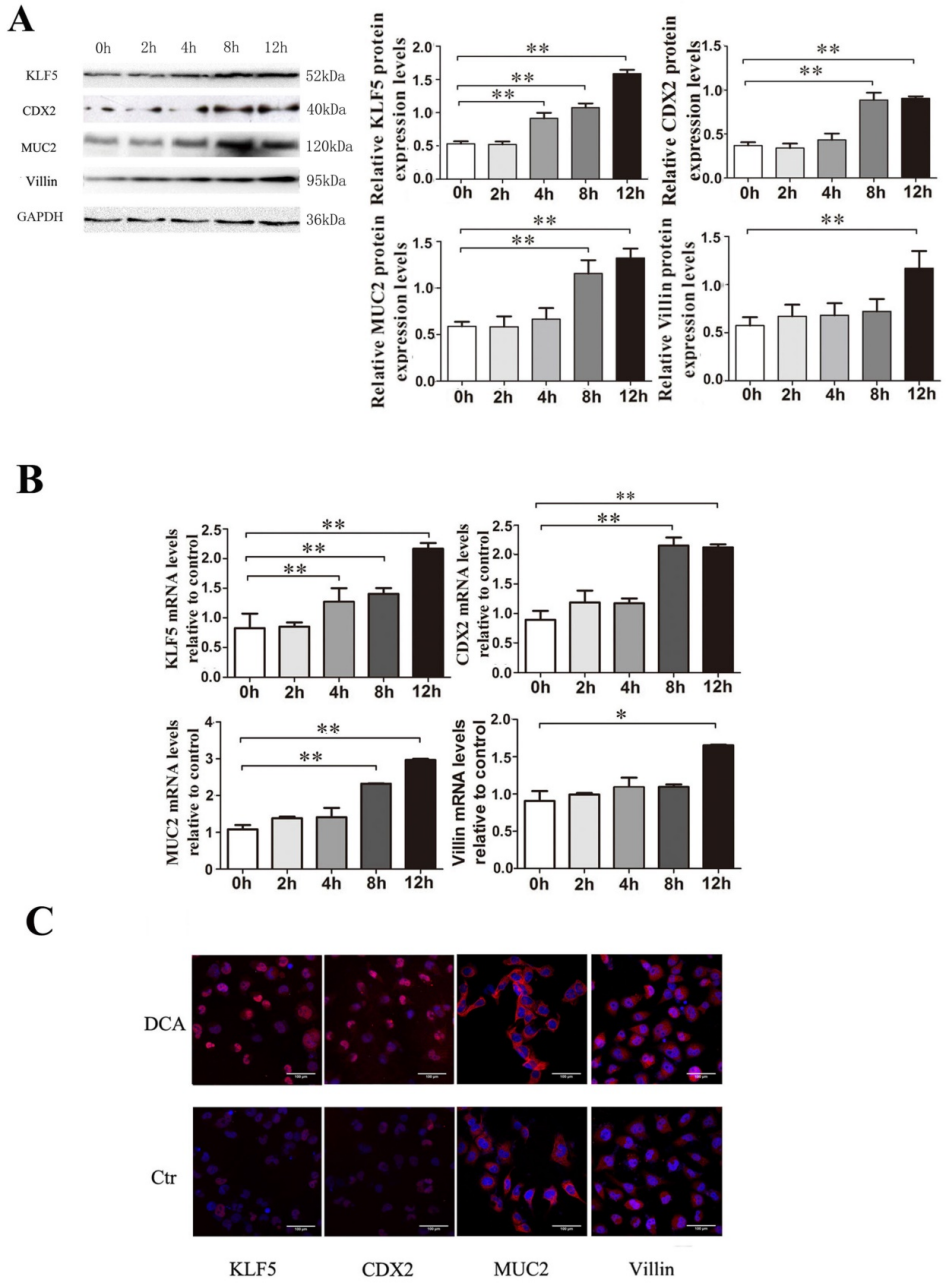
** Effects of bile acids on transcriptional activation of KLF5, CDX2, MUC2 and villin in Het-1A cells.** Het-1A cells were exposed to 200µM DCA for 2, 4, 8 and 12 hours and the effects on the expression of KLF5, CDX2, MUC2 and villin were examined. (A) expressions at protein level. (B)expressions at mRNA levels. (B) determined by immunofluorescence cytochemistry. Results are expressed as the mean ±SEM of three experiments. * p< 0.05, ** p<0.01.

To determine whether KLF5 induction by bile acids occurs during the development of BE, Het-1A cells were exposed to 200 µM DCA for 2, 4, 8 or 12 hours, and examination of effects on the expression of the transcription factor KLF5 at the mRNA and protein levels confirmed that KLF5 expression was augmented by exposure to 200 µM DCA in a time-dependent manner; the intestinal markers Cdx2, MUC2 and villin were also up-regulated simultaneously (Fig. 3).

### KLF5 siRNA inhibits the expression of Cdx2, MUC2 and villin in Het-1A cells

**Figure 4:**
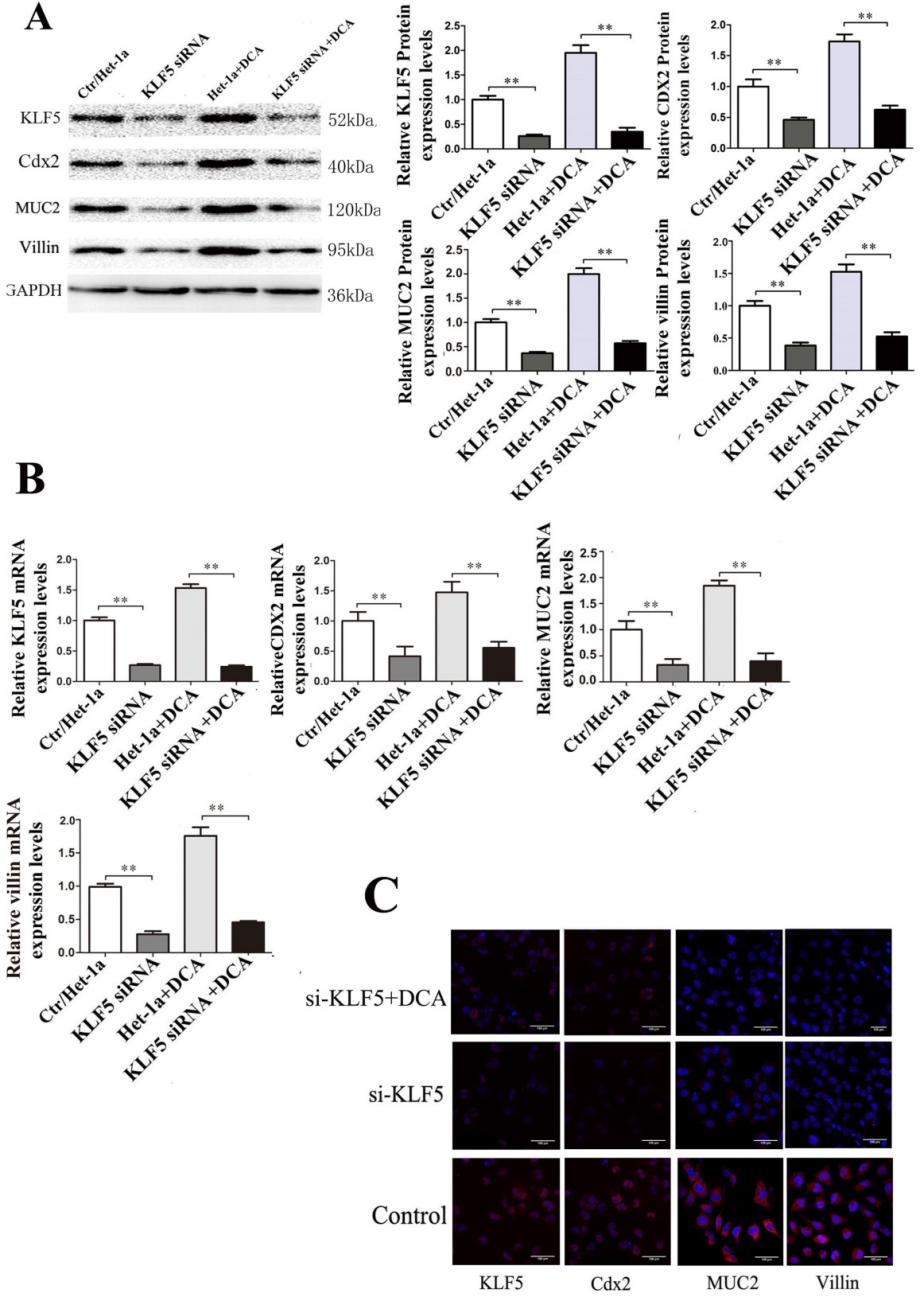
** Effects of KLF5 siRNA on expressions of Cdx2, MUC2 and villin induced by DCA in Het-1A cells.** After KLF5-siRNA, expressions of Cdx2, MUC2 and villin induced by DCA were examined in the Het-1A cells. (A) expressions at protein level. (B)expressions at mRNA levels. (B) determined by immunofluorescence cytochemistry. Results are expressed as the mean ±SEM of three experiments. *p<0.05** p<0.01.

To determine whether Cdx2 and MUC2 expression in villi is induced via activation of KLF5, siRNA approach KLF5 was applied to inhibit endogenous KLF5 expression in Het-1A cells. Results showed that KLF5 siRNA transfection into Het-1A cells significantly inhibited DCA-induced KLF5 expression and reduced the expression of Cdx2, MUC2 and villin (Fig. 4).

### KLF5 transfection up-regulates the expression of Cdx2, MUC2 and villin in Het-1A cells

**Figure 5:**
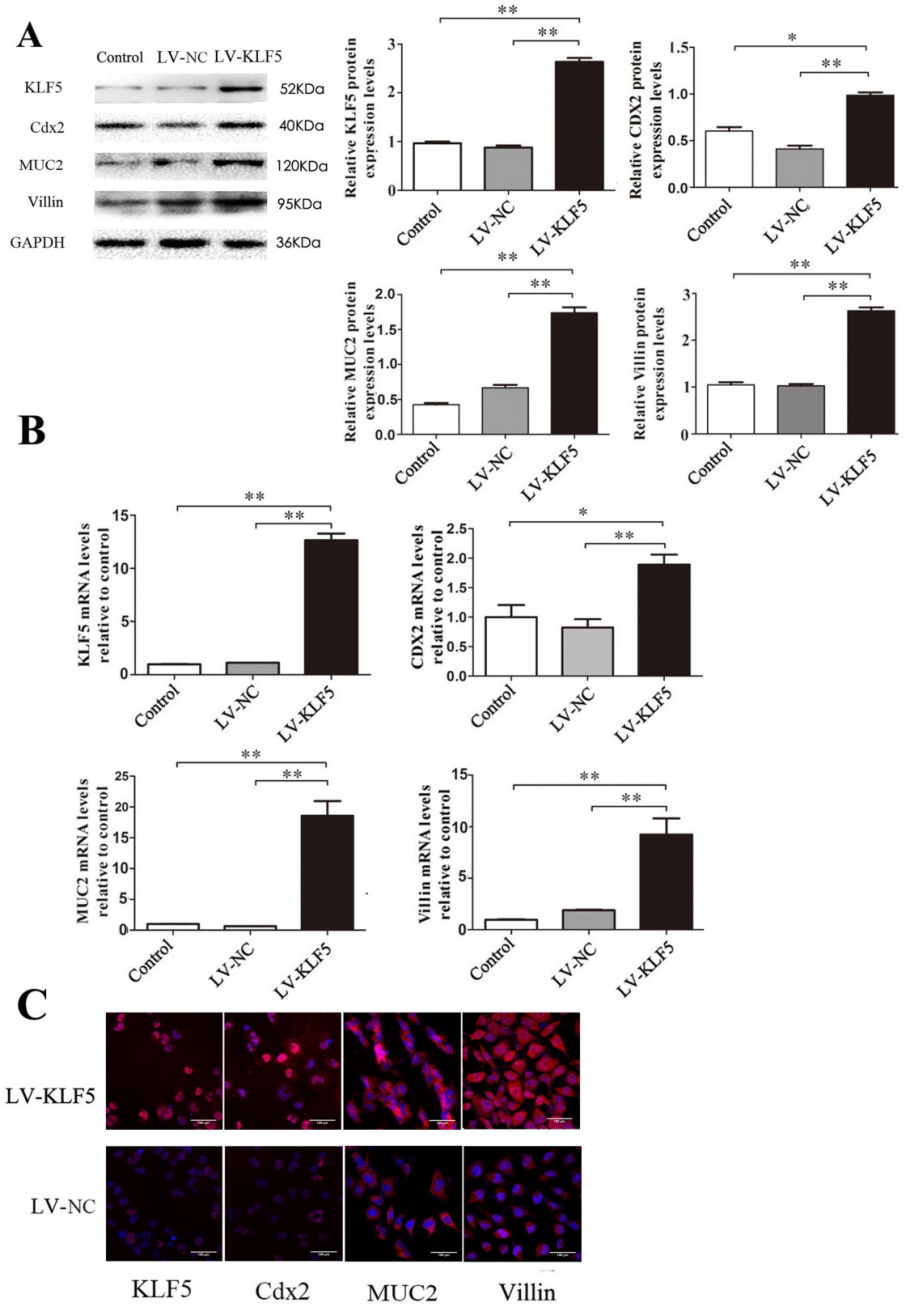
** Effects of KLF5 over-expression on expressions of Cdx2, MUC2 and villin induced by DCA in Het-1A cells.** After transfected with LV- KLF5, the expressions of the Cdx2, MUC2 and villin induced by DCA were carried out in Het-1A cells. (A) expressions at protein level. (B)expressions at mRNA levels. (B) determined by immunofluorescence cytochemistry. Results are expressed as the mean ±SEM of three experiments. *p<0.05, ** p<0.01.

In a lentiviral construct over-expressing KLF5 (LV-KLF5), with LV-NC containing a short noncoding sequence as a control, over-expression of KLF5 was confirmed by western blot after transfection with LV-KLF5. Examination of the effect of over-expression of KLF5 on the expression levels of the three transcriptional factors noted above showed that the expression of Cdx2, MUC2 and villin was significantly increased in the LV-KLF5-transfected cells compared to that in the LV-NC-transfected cells (Fig. 5); this result suggests that KLF5 was sufficient to initiate an intestinal phenotype.

## Discussion

Previous studies have shown that KLF5 interacts with many other transcription factors and regulates the expression of genes involved in cell proliferation, differentiation, angiogenesis and carcinogenesis 20-22. The present study has shown an important modulatory effect of bile acids on KLF5 expression in esophageal squamous cells. In both humans and rats, KLF5 was strongly expressed in BE esophageal squamous cells and was weakly expressed in esophagitis esophageal squamous cells, but no KLF5 expression was detected in normal esophageal squamous cells. KLF5 transfection up-regulated the expression of Cdx2, MUC2 and villin; conversely, KLF5 knockdown attenuated the DCA-induced expression of Cdx2, MUC2 and villin. These findings suggest that DCA mediated the intestinal transdifferentiation of the esophageal squamous epithelium in a KLF5-dependent manner. Thus, in addition to its function as a transcription factor, KLF5 may be linked to an increased risk of BE development.

Clinical observations have shown that GERD, BE and EAC may be a sequential pathological process 23. BE was closely associated with the reflux of gastric and duodenal contents 24, 25. However, the exact outcome of reflux and the true initiator of the conformational changes characteristic of BE have not yet been identified. Bile acids are major constituents of gastroesophageal refluxate and have consistently been found to be associated with increased severity of both esophagitis and BE 8, 26. Initially, the role of bile salts was underestimated, but recent *in vitro* and *in vivo* studies implicated bile salts in the carcinogenesis associated with BE 27, 28. Clinical studies using a Bilitec 2000® apparatus demonstrated that bile-containing reflux is more common and that the bile acid concentrations are significantly higher in patients with BE than in patients with uncomplicated GERD 29. Increased concentrations of bile acids (> 200 μM) have been observed in esophageal aspirates from patients with erosive esophagitis and BE 8, 30. Different bile acids have been shown to have distinct biological effects in terms of carcinogenesis, and their specific effects may be related to their chemical structure and hydrophobic properties 31, 32. It has been found that treatment with acid may reduce the cell growth rate and induce apoptosis, although treatment with bile acid appears to exert a greater influence on cell morphology 33. In the present study, we found that bile acid reflux causes a marked increase in the expression of KLF5, Cdx2, MUC2 and villin. This finding suggests that bile acids may trigger abnormal esophageal squamous cell differentiation, leading to intestinal metaplasia via the KLF5 pathway.

BE metaplasia arises in tissues undergoing continuous regeneration after chronic trauma. Transdifferentiation will occur if the combination of normally expressed transcription factors is altered during the course of regeneration due to mutation or environmental effects. Transdifferentiation can occur in only a few cells, and if the new tissue type has a growth advantage, it will expand to become a macroscopic lesion. Our previous study 34 showed that bile salts up-regulate BMP4 expression, causing mature esophageal squamous cells to change into columnar cells. In the present study, we have shown that the squamous epithelium in the esophagus directly transforms into a columnar epithelial phenotype via KLF5 as a result of exposure to bile acid. This finding suggests that KLF5 may play a role in transdifferentiation of the squamous epithelial phenotype to a columnar epithelial phenotype.

The molecular events responsible for the transdifferentiation of epithelial cells of the esophagus to a columnar cell type are not well understood. KLFs have been shown to be involved in reprogramming somatic cells into inducible pluripotent stem cells and in maintaining the pluripotent state of embryonic stem cells 16,35-37. The expression of Krüppel-like factors is variable according to the specific cell and tissue type; however, KLF5 expression is robust within the gastrointestinal tract, where it predominantly functions as a transcriptional activator 38,39. KLF5 interacts with many other transcription factors and regulates the expression of many genes involved in cell proliferation, differentiation, angiogenesis and carcinogenesis 37,40. Studies of rat and human cells indicate that KLF5 is a component of a network of transcription factors. In the present study, we found that KLF5 was activated in the esophageal mucosa at an early stage of GERD. Therefore, we investigated the effect of KLF5 on the expression levels of CDX2, MUC2 and villin. We found that adenovirus-mediated over-expression of KLF5 increased CDX2, MUC2 and villin expression but that silencing of KLF5 using siRNA resulted in down-regulation of the expression of Cdx2, MUC2 and villin. These results suggest that KLF5 plays an important role in controlling the intestinal transdifferentiation of esophageal squamous cells. Combining this information with previously published data provides additional insight into the molecular events that underlie the transdifferentiation of esophageal squamous cells.

Bile acids activated the expression of Cdx2 in cultured rat esophageal keratinocytes and human esophageal epithelial cells 41,42. Cdx2 may contribute to squamous dedifferentiation during intestinal metaplasia by altering gene methylation 43. Because Cdx2 expression has been detected in BE patients, we sought evidence of Cdx2 expression during the process of transdifferentiation of the esophageal squamous epithelium into a glandular phenotype. Our results demonstrated that bile acid induces Cdx2 protein expression in esophageal squamous cells and promotes a mucinous transdifferentiation process characterized by the secretion of the intestinal mucins, MUC2 and villin. These results suggest that Cdx2 participates in the process of intestinal metaplasia of the esophageal squamous epithelium.

BE appears to result from long-standing chronic, repeated irritation of the esophageal mucosa by gastric and bile acids 44. However, the cellular origin of BE remains controversial, as several different theories of the cellular origin of BE have been proposed 45. Early investigations proposed that cells from the gastroesophageal junction migrate to the distal portion of esophagus as a consequence of damage caused by the refluxate. Other theories implicate abnormal differentiation of pluripotent stem cells in the squamous esophagus or submucosal glands. Finally, BE may evolve from the native squamous epithelium via transdifferentiation as a result of chronic exposure to low pH and bile acids 46. Our results support the transdifferentiation theory, but other theories cannot be excluded, especially the abnormal differentiation of squamous epithelial stem cells into columnar epithelial cells.

In summary, the current study has described a novel molecular mechanism by which bile acid mediates KLF5 gene expression in human and murine esophageal squamous epithelial cells. We have demonstrated that the exposure of the esophageal epithelium to bile acids is an important factor in BE pathogenesis. Further, our results indicate that bile acid induces KLF5 expression in esophageal squamous epithelial cells. Subsequently, the activation of KLF5 up-regulates the expression of CDX2, MUC2 and villin, thereby inducing the intestinal transdifferentiation of esophageal squamous epithelial cells. We conclude that, in addition to its function as a transcription factor, KLF5 may be linked to an increased risk of BE development. Results of this study may contribute to a better understanding of the molecular mechanisms underlying BE pathogenesis.

## Figures and Tables

**Table 1:** Expression of markers in normal, esophagitis and BE esophageal epithelium of human based on IHC

Marker	Expression in normal epithelium (intensity, n, %)	Expression in esophagitis epithelium (intensity, n, %)	Expression in BE epithelium (intensity, n, %)
KLF5	-, 0/20 (0%)	+, 16/59 (27.1%)	++, 18/21 (85.7%)
Cdx2	-, 0/20 (0%)	+, 18/59 (30.5%)	++, 10/21 (100%)
MUC2	-, 0/20 (0%)	+, 27/59 (45.8%)	++, 21/21 (100%)
Villin	-, 0/20 (0%)	+, 12/59 (20.3%)	++, 21/21 (100%)

Expression of KLF5, CDX2, MUC2, Villin was detected by immunohistochemistry in 59 patients. Substantial nuclear expression of KLF5 staining was observed in 85.7% of cases (18/21) of BE. In contrast, KLF5 expression could not be observed in normal esophageal squamous epithelium (0/20). Weak expression (+1) was observed in the basal layers of squamous epithelium in the esophagitis (16/59, 27.1%). IHC, immunohistochemistry; BE, Barrett's esophagus; ++, strong; +, weak; -, negative. (normal vs esophagitis p=0.01053, normal vs BE p=0.0001118, esophagitis vs BE p=0.0001271).

**Table 2:** Histopathological findings at 6 months after surgery.

Histopathologic findings	Esophagojejunostomy (n =20)	Esophago-gastrojejunostomy (n = 24)	Control (n = 10)
Hyperkeratosis	10	14	0
Squamous hyperplasia	10	16	0
Esophagitis	19	21	0
Ulceration	3	4	0
Barrett's esophagus	4 (20%)	5 (20.9%)	0

BE did not occur in control rats, however, 4 of 20 (20%) rats from duodenoesophageal reflux group and 5 of 24 (20.9%) rats in duodenogastroesophageal reflux group developed columnar metaplasia with goblet cells. No obvious difference was found in the incidence of esophageal disease between duodenoesophageal reflux group and duodenogastroesophageal reflux group.

**Table 3:** Expression of KLF5 and intestinal markers in normal, esophagitis and BE of rat esophageal epithelium based on IHC

Marker expression	Normal epithelium (n, %)	Esophagitis epithelium (n, %)	BE epithelium (n, %)
KLF5	-, 0/10 (0%)	+, 12/40 (30.0%)	++, 7/9 (77.7%)
Cdx2	-, 0/10 (0%)	+, 13/40 (32.5%)	++, 9/9 (100%)
MUC2	-, 0/10 (0%)	+, 17/40 (42.5%)	++, 9/9 (100%)
villin	-, 0/10 (0%)	+, 7/40 (17.5%)	++, 9/9 (100%)

Stepwise increased expressions of KLF5, Cdx2, MUC2 and Villin through normal esophageal squamous epithelium, esophagitis and to BE in our rat model. IHC, immunohistochemistry; BE, Barrett's esophagus; ++, strong; +, weak; -, negative. (normal vs esophagitis p=0.009463, normal vs BE p=0.0004483, esophagitis vs BE p=0.0001591).
